# Body Dysmorphic Disorder: Gender differences and prevalence in a Pakistani medical student population

**DOI:** 10.1186/1471-244X-8-20

**Published:** 2008-04-09

**Authors:** Ather M Taqui, Mehrine Shaikh, Saqib A Gowani, Fatima Shahid, Asmatullah Khan, Syed M Tayyeb, Minahil Satti, Talha Vaqar, Saman Shahid, Afreen Shamsi, Hammad A Ganatra, Haider A Naqvi

**Affiliations:** 1 Medical College, The Aga Khan University, Karachi, Pakistan; 2 Section of Psychiatry, Department of Medicine, The Aga Khan University, Karachi, Pakistan

## Abstract

**Background:**

Body dysmorphic disorder (BDD) is a psychiatric disorder characterized by a preoccupation with an imagined or slight defect which causes significant distress or impairment in functioning. Few studies have assessed gender differences in BDD in a non clinical population. Also no study assessed BDD in medical students. This study was designed to determine the point prevalence of BDD in Pakistani medical students and the gender differences in prevalence of BDD, body foci of concern and symptoms of BDD.

**Methods:**

The medical students enrolled in a medical university in Karachi, Pakistan filled out a self-report questionnaire which assessed clinical features of BDD. BDD was diagnosed according to the DSM-IV criteria.

**Results:**

Out of the 156 students, 57.1% were female. A total of 78.8% of the students reported dissatisfaction with some aspect of their appearance and 5.8% met the DSM-IV criteria for BDD. The male to female ratio for BDD was 1.7. Regarding gender differences in body foci of concern, the top three reported foci of concern in male students were head hair (34.3%), being fat (32.8%), skin (14.9%) and nose(14.9%), whereas in females they were being fat (40.4%), skin (24.7%) and teeth (18%). Females were significantly more concerned about being fat (p = 0.005). Male students were significantly more concerned about being thin (p = 0.01) and about head hair (p = 0.012).

**Conclusion:**

BDD is fairly common in our medical student population, with a higher prevalence in males. Important gender differences in BDD symptomatology and reported body foci of concern were identified which reflected the influence of media on body image perception. The impact of cultural factors on the prevalence as well as gender differences in BDD symptomatology was also established.

## Background

Body dysmorphic disorder (BDD) is a psychiatric disorder characterized by a preoccupation with an imagined or slight defect. It is an underrecognized yet relatively common and severe mental disorder that occurs globally [1].

Some degree of concern over physical appearance is quite normal. However, when these concerns with physical appearance reach an intensity where it causes significant subjective distress to the individual and causes impairment in social and occupational functioning and when the perceived appearance flaw is actually nonexistent or slight, it constitutes a disorder: body dysmorphic disorder [2]. This is in concordance with the definition of BDD in the DSM-IV [3]. The DSM IV definition also requires that the preoccupation with the perceived defect must not be better explained by another psychiatric illness like anorexia or bulimia nervosa.

In addition to the concerns about appearance, BDD is marked by time-consuming repetitive compulsive behaviors (mirror checking, excessive grooming behaviours, measuring or comparing the perceived defect), and avoidance (of social situations, mirrors, posing for photographs, bright lights) [4,5]. Studies show that associated occupational and social disability are severe, including absenteeism, poor productivity, unemployment, and marital dysfunction [5,6]. Individuals with BDD have a poor quality of life, are socially isolated, depressed and at a high rate of committing suicide [7].

Studies and surveys have shown that dysmorphic concerns and body image dissatisfaction is increasing in the population [8,9]. BDD has been found to be more prevalent in student populations as compared to community samples [10-13]. To the best of our knowledge, gender differences have been explored extensively in three studies [14-16]; all three were done on clinical samples. Gender differences have not been covered adequately in non-clinical samples.

It is important to discern whether doctors have any element of body image disturbance, because this may have some impact on their practice and specifically, their perception of a patient's physical defects. Given the chronic nature of BDD and the early age of onset (adolescence) [5,17,18], it is highly likely that medical students with body image disturbance, will retain it when they start their professional career.

It is well-recognized that in some communities, being physically unattractive is considered more of a social liability for women than for men [19]. Beauty is a central component of the female gender role stereotype, and women's bodies are more likely to be regarded in an evaluative manner [20]. In the Pakistani culture, physical appearance is a major determinant of the manner in which a female is judged in society. When physical attractiveness affects the value attributed to an individual, the desirability of a physically attractive appearance increases and the risk of an individual developing body image concerns become more likely. This phenomenon has been exemplified in cross-cultural studies which showed that Americans who consign greater value to physical attractiveness are more likely to develop body image concerns than Asians and Germans [12,21,22]. In light of this background, we hypothesized that the prevalence of BDD would be higher in females and they would report different foci of concern compared to males. Given the fact that society has high expectations from doctors in terms of grooming and appearance, we hypothesized that the prevalence of BDD would be higher in our medical student population than other student samples.

This study was conducted to determine the prevalence of BDD in medical students and the gender differences in prevalence of BDD, body foci of concern and symptoms of BDD.

## Methods

### Study design and study site

This cross sectional survey was conducted among medical students of the Aga Khan University (AKU), a private educational university with its own teaching hospital, in Karachi, Pakistan. The medical students in the medical college come from different cities and towns all over Pakistan.

### Study sample, selection criteria and data collection

At the time of study, a total of 450 students divided over 5 years, were enrolled at the medical college. We required a sample size of 160 subjects to fulfill the objectives of our study at a 95% confidence level. This sample size was calculated assuming a 13% prevalence of BDD, 5.5% bound-on error, and 10% non-response rate. The prevalence value of 13% was chosen because it is the maximum value of BDD reported in a sample of college students, using the DSM IV criteria [23].

All medical students studying in the five years of the medical college were eligible for participation. Our exclusion criteria were: students who submitted incomplete forms and students who reported a diagnosis of anorexia nervosa or bulimia nervosa.

Stratified sampling was done, with equal distribution of questionnaires to each of the five years. Informed consent was taken. The nature of the study and the right to withdraw was fully explained to the participants. The participants were requested to fill out the questionnaire honestly. An opaque black box was used as a drop-in box. The students were requested to return the questionnaires within three days. Strict confidentiality was ensured. The study was conducted in compliance with the 'Ethical principles for medical research involving human subjects' of the Helsinki Declaration. The study protocol was discussed with supervising faculty for possible ethical concerns.

### Questionnaire

Our questionnaire comprised of three major parts: the first part covered demographic information, the second part incorporated a pre-tested questionnaire to measure BDD and the last part addressed symptoms of BDD.

#### Part 1

Demographic information consisted of four parameters: age, gender, class of enrollment in medical college and marital status.

#### Part 2

A structured questionnaire, which has been successfully tested for validity and reliability by Cash et al. [24], was adapted with permission from the author. The study clearly supported the reliability and validity of the 7-item Body Image Disturbance Questionnaire (BIDQ) to measure "body image disturbance" in a non clinical population (college students). It is important to clarify the meaning of "body image disturbance" and its relation to BDD.

There are two terms related to body image which are present in research literature: body image disturbance and body image dissatisfaction. They are two different entities. While dissatisfaction with some aspect of one's appearance increases one's risk for experiencing emotional distress and functional impairment, dissatisfaction itself does not constitute a disorder. Individuals may be dissatisfied with their general appearance or a particular aspect, yet the impact of this negative body image evaluation on daily functioning can range from minimal to extreme. Thus, body image disturbance is not merely body image dissatisfaction [24].

Both Thompson et al. and Cash et al. have opted for a multidimensional definition of body image disturbance [25,26]. In 1992, Thomas et al. proposed a new DSM IV category: Body Image Disorder, which encompassed body image disturbance [26]. In 1999, Thompson et al. [27], proposed a definition for body image disturbance that entails "a persistent report of dissatisfaction, concern, and distress that is related to an aspect of appearance. ... and some degree of impairment in social relations, social activities, or occupational functioning. ...". This perspective explicitly expresses the contemporary definition of BDD in DSM IV [3].

In comparison to currently existing validated instruments that measure a specific dimension of body image, the BIDQ is more comprehensive in its brief but integrative assessment of body image disturbance [24]. Also, unlike other questionnaires, the BIDQ was designed to assess body image disturbance or BDD in a non clinical population. BDD lies at the extreme end of the body image disturbance spectrum.

After adaptation of the questionnaire, it was pre-tested on a group of students to screen for potential problems. No changes were made after conducting the pre-test. [See questionnaire in Additional File 1]. Each question in the 7-item BIDQ had responses in the form of a 5 point Likert scale. All seven questions assessed responses according to the DSM-IV criteria [3]. The first and second question assessed the level of concern/preoccupation with the physical defect, the third assessed the level of subjective distress and the rest assessed the level of impairment in social, educational and occupational functioning. As indicated in the scoring manual, the score was the mean of the seven items scaled from 1 to 5. A score greater than 3.0 was taken as the cut-off for diagnosing BDD. According to Cash, a score of 3.0 or more will detect 98% of individuals with BDD. Someone who scores over 3.0 will have a mid-point value per question over three. A mid-point value over three per question implies that the person satisfies the DSM-IV criteria for BDD. The fourth and fifth response to each question represents the level of severity which meets the DSM-IV criteria for BDD [See questionnaire in Additional file 1].

BDD lies at the extreme end of the body image disturbance spectrum and the graded responses allow one to identify BDD.

#### Part 3

The six symptoms which were addressed were: 1. Habit of compulsive mirror checking or glancing at image in reflective surfaces, 2. Compulsively touching the physical "defect", 3. Trying to hide or conceal the physical "defect", 4. Measuring the physical "defect" against people around, 5. Comparing the physical "defect" with people in magazines or on television, 6. Avoidance of doing certain things like looking into a mirror or getting photographed.

### Statistical analysis

Data was entered in Epi Data version 3.1 and analyzed in Statistical Package for Social Sciences version 14.0 (SPSS, Inc., Chicago, IL, USA). Descriptive statistics were performed. Results were recorded as frequencies, means ± standard deviations (SD) and p-values. The Chi-square test and Fisher's exact test were used for univariate analysis of categorical variables. Tables and figures were used for comprehensive viewing of the results. For all purposes, a p-value of < 0.05 was taken as the criteria of significance.

## Results

A total of 190 questionnaires were distributed and 168 students (response rate of 88.4%) returned the forms. After accounting for the exclusion criteria, 156 students were qualified for analysis. There were 67 (42.9%) male and 89 female (57.1%) students. The mean age of both sexes was similar (20.8 years ± 2 vs 20.5 years ± 1.8). Most of the students were unmarried (98.7%). Out of the 156 students, 123 (78.8%) were dissatisfied with some aspect of their physical appearance. More females were dissatisfied with some aspect of their physical appearance than males (88.8% vs 76.1%).

The prevalence of BDD was computed to be 5.8% (9/156). The male to female ratio was 1.7 (7.5%:4.5%). Three out of the 5 male students, and 3 out of the 4 female students, reported their focus of concern as being fat.

**Figure 1:**
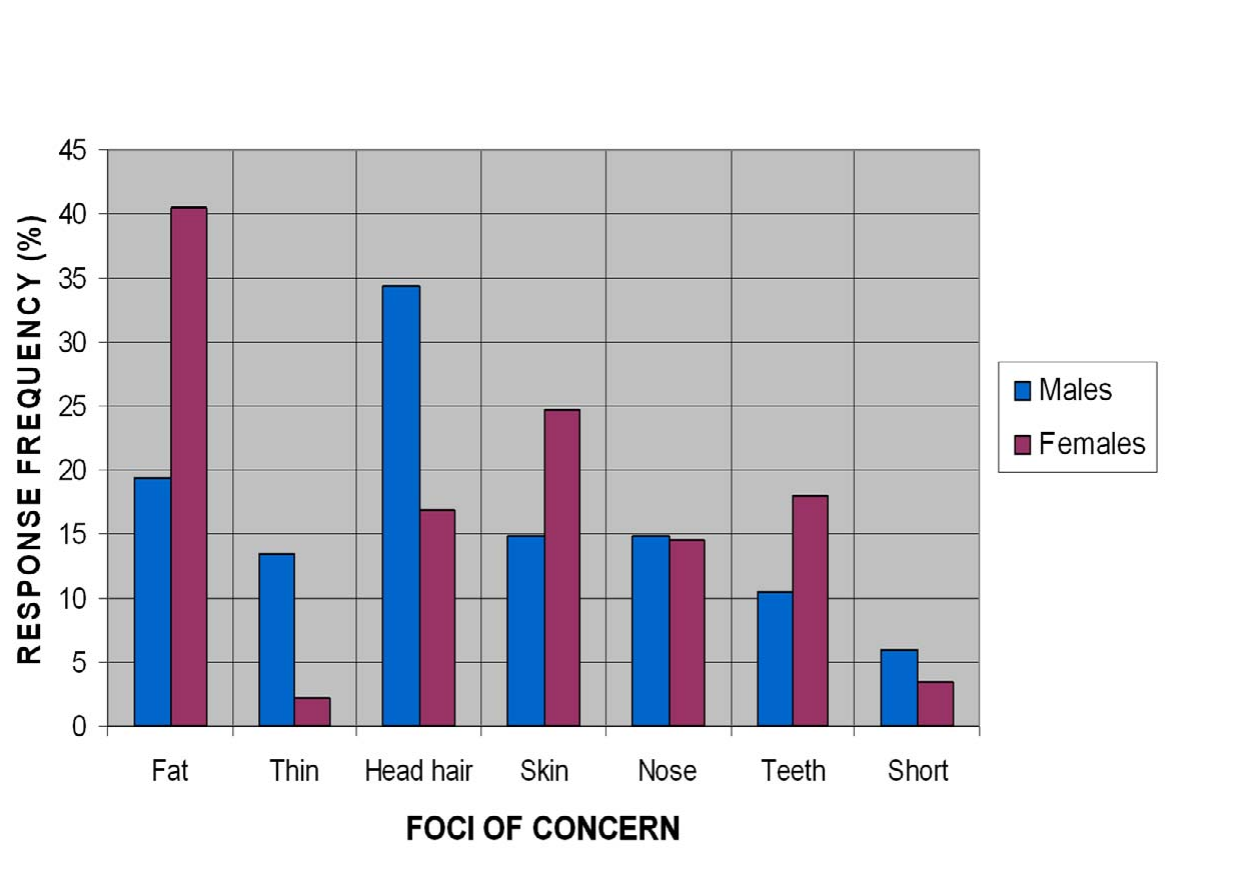
Comparison of selected foci of concern among male and female students.

Table 1 shows the foci of concern of all students and compares them between male and female students. The top three reported body foci of concern of the students were: being fat (31.4%), head hair (24.4%) and skin (20.5%), in that order. When comparing gender, females were found to be significantly more concerned about being fat (p value = 0.005). Male students were found to be significantly more concerned about being thin (p value = 0.010) and about head hair (p value = 0.012). The top three reported body foci of concern in male students were: head hair (34.3%), being fat (32.8%), skin (14.9%) and nose (14.9%). The top three reported body foci of concern in female students were: being fat (40.4%), skin (24.7%) and teeth (18%). Figure 1 shows a comprehensive cluster bar chart which compares selected foci of concern between male and female students.

**Table 1:** Comparison of foci of concern among male and female subjects

BODY FOCI OF CONCERN*	**TOTAL (n = 156)**	**MALES (n = 67)**	**FEMALES (n = 89)**	**p-value**^**§**^
	**Number (%)**	**Number (%)**	**Number (%)**	
		
Body weight	60 (38.5%)	22 (32.8%)	38 (42.7%)	0.210
Fat	49 (31.4)	13 (19.4%)	36 (40.4%)	0.005
Thin	11 (7.1%)	9 (13.4%)	2 (2.2%)	0.010
Head hair	38 (24.4%)	23 (34.3%)	15 (16.9%)	0.012
Skin	32 (20.5%)	10 (14.9%)	22 (24.7%)	0.134
Nose	23 (14.7%)	10 (14.9%)	13 (14.6%)	0.956
Teeth	23 (14.7%)	7 (10.4%)	16 (18%)	0.189
Being short	7 (4.5%)	4 (6%)	3 (3.4%)	0.464
Other**	23 (14.7%)	9 (13.4%)	14 (15.7%)	--

* These foci have multiple responses and percentages in columns will not add up to 100%.

** Other foci of concern included eyes, ears, thighs, buttocks, genitalia, body hair, etc but none of these foci reached 4% of total independently.

^§ ^Chi-square test was applied here to find significant differences in foci of concern among male and female subjects.

Table 2 compares the responses of male and female students to six questions which addressed symptoms of BDD. A large proportion of students (23.1%) reported that they compulsively checked their image in mirrors, very often. The distribution of responses to most questions was similar across gender. However, significantly more females (p value = 0.009) compared their perceived physical "defect" with people on television, than males.

**Table 2:** Frequency of reported symptoms in male and female students

RESPONSES TO QUESTIONS ADDRESSING SYMPTOMS*	**TOTAL (n = 156)**	**MALES (n = 67)**	**FEMALES (n = 89)**	**p-value**^**§**^
	**%**	**%**	**%**	
		
Q1. Are you in a habit of compulsive mirror checking or compulsively glancing at your image in reflective surfaces (e.g. windows, doors)?				
1. Never	20.5	25.4	16.9	0.419
2. Occasionally to moderately often	56.4	52.2	59.6	
3. Very often to extremely often	23.1	22.4	23.6	
Q2. Do you compulsively touch your physical "defect"?				
1. Never	46.2	46.3	46.1	0.063
2. Occasionally to moderately often	42.9	49.3	38.2	
3. Very often to extremely often	10.9	4.5	15.7	
Q3. Have you tried to conceal/hide your physical "defect"? (e.g. make up, scarves, clothing, beard)				
1. Never	40.4	50.7	32.6	0.057
2. Occasionally to moderately often	44.2	34.3	51.7	
3. Very often to extremely often	15.4	14.9	15.7	
Q4. Have you ever measured your physical "defect" against people around you?				
1. Never	26.9	31.3	23.6	0.153
2. Occasionally to moderately often	54.5	56.7	52.8	
3. Very often to extremely often	18.6	11.9	23.6	
Q5. Have you ever compared your physical "defect" with people in magazines or on television?				
1. Never	23.1	34.3	14.6	**0.009**
2. Occasionally to moderately often	53.2	41.8	61.8	
3. Very often to extremely often	23.7	23.9	23.6	
Q6. Do these concerns about your physical "defect" make you avoid doing certain things? (e.g. looking into a mirror, getting photographed, avoiding social gatherings)				
1. Never	46.2	46.3	46.1	0.888
2. Occasionally to moderately often	47.4	46.3	48.3	
3. Very often to extremely often	6.4	7.5	5.6	

* In the questionnaire, each question had 5 graded responses: 1) Never 2) Occasionally 3) Moderately often 4) Very often 5) Extremely often. These 5 responses have been collapsed to 3 to allow better interpretation.

^§ ^Chi-square was applied here to find significant differences in foci of concern among male and female subjects.

## Discussion

The present study revealed that the prevalence of BDD in the medical students in the medical college was 5.8% with a male to female ratio of 1.7. The top three reported body foci of concern of the students were: being fat, head hair and skin. When comparing across gender, females were found to be significantly more concerned about being fat whereas male students were found to be significantly more concerned about being thin and about head hair.

### Body Image Dissatisfaction

Body image dissatisfaction was quite high in the medical students in our study. The majority (78.8%) of the students reported that they were concerned about some aspect of their appearance. This is slightly higher than the levels of body image dissatisfaction reported in the study by Bohne et al [12] on American college students (74.3%) and Fitts et al [28] on college students (70%). Our reported level of body image dissatisfaction was much higher than that in a female Turkish college student sample (43.8%) [10].

### Prevalence of BDD

Table 3 compares the point prevalence of BDD in studies done in non-clinical samples. It can be seen that the prevalence of BDD in our sample (5.8%) is higher than that in the three other college student samples: German students (5.3%), American students (4%) and Turkish students (4.8%). These three studies are the most comparable because the sample is a non clinical one (college students) and the students have a similar mean age. However, the samples were ill balanced as per the gender distribution to compare the male to female ratio.

**Table 3:** Comparison of studies assessing the prevalence of BDD

**AUTHOR**	**POPULATION DETAILS**	**PREVALENCE OF BDD**
**STUDENT SAMPLES**		

Bohne A et al. [11]	German college students (n = 133, 73.7% females, mean age = 22)	5.3%
Bohne A et al. [12]	American college students (n = 101, 82.2% females, mean age = 21)	4%
Cansever A et al. [10]	Turkish college students (n = 420, 100% females, mean age = 19)	4.8%
Biby et al. [23]	Undergraduate students (n = 102, 76.5% female)	13%
Sarwer et al. [43]	American college students (n = 559, 100% females)	2.5%
Taqui A M et al. (present study)	Pakistani medical college students (n = 156, 57.1% females, mean age = 21)	5.8%

**COMMUNITY/POPULATION BASED SAMPLES**		

Otto M et al. [13]	Boston community sample (n = 976, 100% female, age = 36–44)	0.7%
Faravelli C et al. [31]	Italian community sample (n = 673, 100% female)	0.7%
Rief W et al. [44]	German population based survey (age 14–99)	1.7%

The slightly higher prevalence of BDD in our sample could be accounted for by a number of different factors. Medical students might be more conscious about their physical appearance than students in most other fields of study, because of society's high expectations from a doctor in terms of grooming and appearance. An association between BDD and education/occupation in art and design has been shown [29]. However, it is debatable whether an education in art and design may be a contributory factor to the development of BDD or if patients with BDD tend to have an interest in aesthetics. Similarly, the medical profession could be acting as a contributory factor to the development of BDD.

Alternatively, the higher prevalence of BDD could also reflect cross-cultural differences in the value placed on physical attractiveness and the resulting socio-cultural pressures.

As seen from Table 3, the prevalence of BDD in our sample was much higher than that in community based or population samples (5.8% vs. 0.7–1.7%). One plausible explanation is that since the community samples included a large proportion of people above the age of 30, the lower prevalence (0.7%–1.7%) reflects only those people in whom BDD has persisted into late adulthood.

### Gender ratio for prevalence of BDD

The present study showed that the male to female ratio for BDD it was 1.7. Comparable studies on non-clinical samples do not show a consistent ratio. Our value was similar to a community study from the United States (n = 373) which found that BDD was present in 1.2% of men and 1% of women, giving the male to female ratio to be 1.2 [30]. However, a community study from Florence, Italy (n = 673), revealed that 1.4% of women, but no men, had BDD [31]. The comparable studies done on college student samples either had predominantly female populations or had too small a sample size to give a conclusive male to female ratio for BDD (See Table 3) [10-12].

In clinical sample populations, the gender ratio has shown great variability. Three studies contained more men than women [6,16,32], three contained more women than men [15,33,34] and two contained nearly equal proportions of men and women [5,14,35]. However, these studies were subject to the bias of convenience sampling and do not reflect the true gender ratio in the community. It can be cautiously concluded that the gender ratio in non-clinical populations is not known and may exhibit variability in different populations. These inconsistent gender ratios in both clinical and community samples highlight the need to examine the prevalence of BDD in women and men in larger epidemiological studies.

Our finding of the male to female ratio being 1.7 for BDD was inconsistent with our hypothesis that more females would have self-reported BDD. One plausible reason could be that in medical school, the primary pressure for increased consciousness of self-appearance is society's high expectations from doctors in terms of appearance. This might overshadow the other factor that physical appearance is a means for evaluation of females in the Pakistani society. Hence, the prevalence of self-reported BDD was not higher in females. It is interesting to note that although more females reported body image dissatisfaction than males (88.8% vs 76.1%), the prevalence of BDD was lower in females.

### Gender differences in reported body foci of concern

We found four studies which analyzed gender differences extensively. Three of these studies looked at clinical samples [14-16] and one looked at a non clinical sample [24]. Cash et al did a study on a young college population in the US. Out of the three studies which looked at clinical samples, two studies were done in the US and one was done in Italy.

The present study revealed that the most frequent foci of concern were being fat (31.4%), head hair (24.4%), skin (20.5%), nose (14.7%) and teeth (14.7%). Collectively, this is consistent with the findings in most studies which say that body shape, skin and facial features are among the most common foci of concern [14,15,25,35-37].

Regarding gender differences, Table 1 and Figure 1 show that females were more concerned about being fat, their skin and teeth. Males were more concerned about being thin, being short and their head hair. However, univariate analysis revealed that females were significantly more concerned about being fat and male students were significantly more concerned about being thin and their head hair.

Our findings were concurrent with those in the study done on the US college population [24], which revealed that the most frequent foci of concern for both genders were weight/shape related concerns, followed by facial features and muscularity for males and legs/thighs and facial features for females. The US study did not elaborate on the weight/shape concerns as being fat or thin. One notable difference is that in the study a sizable proportion of females expressed legs, thighs and breasts as foci of concern, whereas in our study, these responses accounted for less than 4% and were not listed individually in Table 2. This is not surprising. There is a credible reason for this discrepancy, being the fact that in Pakistan, which is a conservative Muslim country, females expressing concern over the size of legs/thighs and breasts, is considered a taboo subject. Females could be hesitant in reporting these foci of concern even if they were preoccupied with them, resulting in the discrepancy.

Looking at the three studies done on clinical samples, some of this study's findings were consistent with the two studies done in the US [14,15]: males were more likely to be concerned with thinning hair and small body built, whereas women were more likely to be preoccupied with their weight. However, it did not confirm the findings that women were more concerned with their hips and excessive body hair. Our findings that males were more concerned about their height, was consistent with the study done in Italy [16].

In all the four studies, it was seen that males were more likely to be preoccupied with their genitals. However, our study did not confirm this finding. Again, societal taboos could lead to underreporting of this body focus of concern.

On the whole, the gender similarities and differences in reported body foci of concern were similar to previous studies. The only major discrepancies (females focusing more on legs/thighs and breasts and males focusing on genitals, in other studies) are explained by the socio-cultural norms in our country.

It is not surprising to see that our finding of females being more concerned about being fat and males being more concerned about being short/body size and head hair, are reflected by appearance concerns which are commonly displayed in advertisements and media. It is well recognized that the media is portraying a steadily thinning ideal body image for women [38,39] and a well-built, muscular body image as an ideal for men [40,41]. A high proportion of students (76.1%) in our study reported that they compared their perceived physical "defect" with people on television. This suggests that the media plays a major role in determining the ideal body image which a high proportion of individuals strive to attain.

### Symptoms of BDD

Our study showed that the symptoms of BDD were fairly common in our sample. However, the severity of majority of the symptoms was not extreme. Table 2 shows the responses of both male and female students to the questions which addressed BDD symptoms. A large proportion of students (79.5%) had the habit of checking their image in reflective surfaces at least occasionally. Twenty three percent of the students practiced this act very often or extremely often. About 60% of the students tried to camouflage their perceived physical "defect" and 54% had the habit of compulsively touching their physical "defect". It was interesting to see that a large proportion of students (73.1%) measured their physical "defect" against people around them and 76.9% compared their physical "defect" with people in magazines or on television.

On the whole, the severity of symptoms was similar across gender. However, it was found that significantly more females (p value = 0.009) compared their perceived physical "defect" with people on television, than males. 85% of females compared their perceived physical "defect" with people on television compared to 65% of females. It is known that the perceived body image of females is directly affected by advertising and media programmes which emphasize the pursuit of a thin body image [42].

### Limitations

The present study had a number of limitations which merit discussion. The major limitation in our study was the use of a questionnaire which had not been validated in our population. The BIDQ has been validated in an American population. At the time of study, there were no instruments validated for use in our population. In these circumstances, we chose the BIDQ over other instruments because it is the only one which has been designed to assess BDD in college students, a non-clinical population. We believe that the high psychometric properties of the BIDQ would not be much altered even if it was used without a validation study. As mentioned above, with the cut-off of 3, the BIDQ is very sensitive at picking up BDD.

It is known that body image problems are more common in young people when BDD may be less severe. They are more common in women and overlap with sub-clinical eating disorders. The finding that 31.4% of students reported being fat as the focus of concern may be reflecting this. In addition to detecting BDD, the BIDQ can capture body image disorders including eating disorders. To exclude these individuals, the study questionnaire included a question which asked students whether they had been diagnosed with anorexia nervosa or bulimia nervosa. However, a single question screen is unlikely to be very effective. Therefore, the prevalence of BDD in this study might be overestimated or it is possible that some of the students with milder BDD had an eating disorder.

Since our data was based on self report, there was no objective way to know whether the defects perceived by the students who appeared to meet the criteria for BDD, were exaggerated or not. This may affect the reliability of our results. The present study was done on medical students from one institution only and this somewhat restricts the generalization of the results to the whole medical student population in Pakistan. However, our sample is likely to be representative since the enrolled students in the university are from all over Pakistan.

In light of these limitations, the findings of the study must be interpreted in a prudent manner.

## Conclusion

BDD is fairly common in our medical student population. Our study indicates that BDD is more prevalent in males. However, studies with larger sample sizes are required to confirm this. The study also delineates the gender differences in BDD symptomatology and reported body foci of concern. When comparing gender, females were significantly more concerned about being fat and males were significantly more concerned about being thin and their head hair. These reported body foci of concern reflected the influence of media on body image perception.

Previous studies had highlighted the paucity of literature on BDD in non-Western cultures [36]. We have addressed that need and demonstrated the influence of socio-cultural norms on body image concerns. Cultural factors influence the prevalence as well as gender differences in BDD symptomatology. Further research is required to establish the gender ratio in the prevalence of BDD in community settings. Cross-cultural studies are required to establish the influence of sociocultural background on the prevalence of BDD.

## Competing interests

The author(s) declare that they have no competing interests.

## Authors' contributions

AMT and MS conceived the study. AMT, MS and FS were involved in the design of the study. All authors were involved in data collection. AMT, AK and SAG were involved in the data analysis and data interpretation. AMT prepared the manuscript. HAN provided critical feedback and guidance and was responsible for the study's ongoing management. All authors read and approved the final manuscript.

## Pre-publication history

The pre-publication history for this paper can be accessed here:



## Supplementary Material

Additional file 1 Questionnaire. Questionnaire with scoring method. Click here for file
