# Supplementary material for: Body Dysmorphic Disorder: Gender differences and prevalence in a Pakistani medical student population
Source: BMC Psychiatry. 2008 Apr 9;8:20. doi: 10.1186/1471-244X-8-20 (PMC2329619; doi:10.1186/1471-244X-8-20)
Supplement: Additional file 1 — Questionnaire. Questionnaire with scoring method. [file 1471-244X-8-20-S1.pdf]

PART 2 of the questionnaire

1. Are you concerned about the appearance of some part(s) of your body, which you consider especially unattractive?
   1. □ Not at all concerned
   2. □ Somewhat concerned
   3. □ Moderately concerned
   4. □ Very concerned
   5. □ Extremely concerned

2. Which aspect/feature (e.g. skin, hair, nose, teeth, body size: thin or fat etc) .You can list more than one. ____

3. If you are at least somewhat concerned, do these concerns preoccupy you? That is, you think about them a lot and they're hard to stop thinking about?
   1. □ Not at all
   2. □ Somewhat preoccupied
   3. □ Moderately preoccupied
   4. □ Very preoccupied
   5. □ Extremely preoccupied

4. Has your physical "defect" caused you a lot of distress, torment, pain or difficulty? How much?
   1. □ No distress
   2. □ Mild, and not too disturbing
   3. □ Moderate and disturbing, but manageable
   4. □ Severe, and very disturbing
   5. □ Extreme and disabling

5. Has your physical "defect" caused you impairment in your social, occupational or other important areas of functioning? How much?
   1. □ No limitation
   2. □ Mild interference, but overall performance not impaired
   3. □ Moderate, definite interference but still manageable
   4. □ Severe, causes substantial impairment
   5. □ Extreme, incapacitating

6. Has your physical "defect" significantly interfered with your social life? How much?
   1. □ Never
   2. □ Occasionally
   3. □ Moderately often
   4. □ Often
   5. □ Very often

7. Has your physical "defect" significantly interfered with your education or your ability to function in your role? How much?
   1. □ Never
   2. □ Occasionally
   3. □ Moderately often
   4. □ Often
   5. □ Very often

8. Do you ever avoid things because of your physical "defect"? How often?
   1. □ Never
   2. □ Occasionally
   3. □ Moderately often
   4. □ Often

5. □ Very often

NOTES

- All the questions are adapted from the original BIDQ without any modifications except question 2. Question 2 was added by us to assess the foci of concern.
- Question 1 and questions 3-8 were the seven questions of the BIDQ which constituted the scoring scale

SCORING

- The score was the mean of the seven items scaled from 1 to 5. Score for a student = (Sum of responses to the seven questions) / 7.
- A score > 3.0 was the cut-off for identifying BDD.
- For example, if responses to the 7 questions were: 3, 4, 3, 3, 4, 5, and 4; the score would be (3+4+3+3+4+5+4)/ 7 = 3.7.
